# Acute kidney injury: incidence and quality of care in patients with type 2 diabetes

**DOI:** 10.1093/ckj/sfag177

**Published:** 2026-05-30

**Authors:** Simone L Dam, Dion G L de Martines, Kim B Noordhuis, Willemien J Kruik-Kollöffel, Aernoud T L Fiolet, Gozewijn D Laverman, Christina M Gant

**Affiliations:** Department of Clinical Pharmacy, ZGT, Almelo, The Netherlands; Department of Internal Medicine/Nephrology, ZGT, Almelo, The Netherlands; Department of Nephrology and Hypertension, University Medical Center Utrecht, Utrecht University, Utrecht, The Netherlands; Department of Internal Medicine/Nephrology, ZGT, Almelo, The Netherlands; Department of Clinical Pharmacy, ZGT, Almelo, The Netherlands; Department of Cardiology, University Medical Center Utrecht, Utrecht, Utrecht University, Utrecht, The Netherlands; Department of Internal Medicine/Nephrology, ZGT, Almelo, The Netherlands; University of Twente, Biomedical Signals and Systems (BSS) Research Group, Enschede, The Netherlands; Department of Nephrology and Hypertension, University Medical Center Utrecht, Utrecht University, Utrecht, The Netherlands; Department of Internal Medicine/Nephrology, Dianet, Amsterdam University Medical Center, Amsterdam, The Netherlands

To the Editor,

Acute kidney injury (AKI) occurs in one in five hospitalized adults [1] and is associated with increased mortality and risk for chronic kidney disease [2, 3]. Patients with type 2 diabetes (T2D) have an increased risk of AKI [2]. Quality of in-hospital AKI care varies [4], and data on AKI management in patients with T2D remain limited. We therefore evaluated the incidence of AKI and quality of in-hospital AKI care in T2D patients.

This subanalysis was performed in a prospective observational cohort of patients with T2D [the DIAbetes and LifEstyle Cohort Twente (DIALECT)] treated in secondary care in the Netherlands. Data were collected from electronic health care records. AKI incidence was determined using all available routinely collected serum creatinine (SCr) measurements. AKI was defined as an increase in SCr by 26.5 µmol/l within 48 h or an increase of SCr by ≥50% of the last known baseline SCr value up to 1 year before [5]. Quality-of-care indicators for in-hospital AKI management were derived from the 2012 KDIGO guidelines and included nephrotoxic drug discontinuation (within 24 h of AKI onset), optimization of volume status and renal perfusion pressure (within 24 h), SCr monitoring (day following AKI), and performing non-invasive diagnostic workup (anytime during AKI) [5]. Cause of AKI was established by manual case review and categorized as reduced renal perfusion pressure (prenal), either decompensated or after recompensation of heart failure (prerenal-HF), renal, and postrenal. Additionally, documentation of AKI and consultation of internal medicine were recorded. All-cause mortality was assessed as a secondary outcome by comparing patients with AKI with age- and sex-matched patients without AKI. Controls had to be at risk at the time of the matched case’s first AKI episode, which served as the index date.

Among 672 patients included between 2009 and 2019, 149 (22.2%) had ≥1 AKI episode and 106 (15.8%) had ≥1 in-hospital AKI episode. The mean follow-up duration was 7.7 ± 3.2 years and 39.3% of patients were female (Supplementary Table S1). Mean age was 64 ± 10 years. AKI incidence was 3.25 per 100 person-years. The most common AKI cause was prerenal (60.7%), followed by prerenal-HF (26.9%), renal (30.3%), postrenal (3.4%) and unknown (1.4%); multiple causes could contribute to a single episode. Regarding quality of care, occurrence of AKI was documented in 73% and creatinine was monitored in 72% of AKI episodes (Fig. 1). Nephrotoxic drugs were continued in 54% of episodes following AKI. In prerenal AKI, kidney perfusion-affecting drugs were continued in 48% and fluid administration was performed in 44% of AKI episodes. In prerenal-HF, kidney perfusion-affecting drugs were administered in 84% and fluid administration was performed in 8% of AKI episodes. The incidence of all-cause mortality was higher in patients with AKI as compared to those without (14.5 versus 4.0 per 100 person-years, hazard ratio 4.86, 95% CI 2.92–8.11, *P* < .001).

**Figure 1:**
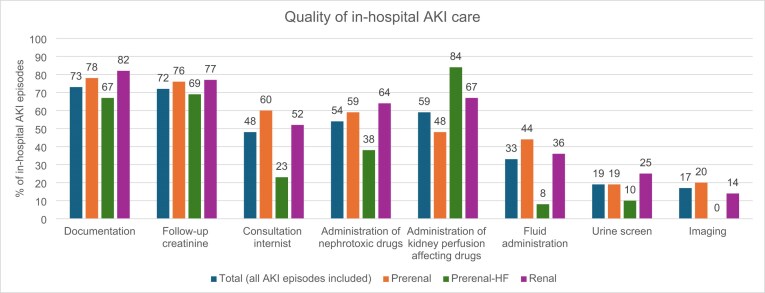
Quality of in-hospital AKI care. “Postrenal” and “unknown” cause not shown separately due to low numbers. Missing values administration of nephrotoxic drugs, kidney perfusion-affecting drugs and fluid (*n* = 5).

AKI remained undocumented in a quarter of patients, raising concerns about its recognition. Moreover, in approximately half of prerenal AKI episodes, kidney perfusion-affecting drugs were continued and fluid administration was lacking within the first 24 h after AKI onset. This is particularly relevant as prerenal etiology represents a common and potentially reversible cause of AKI. The higher post-AKI mortality observed in our cohort underscores the vulnerability of these patients and highlights the need for vigilant monitoring and proactive management.

In this real-world cohort of T2D patients, the incidence of AKI is substantial and quality of in-hospital AKI care is suboptimal. By structured analyses of quality of care, we highlight clear opportunities to improve AKI care: first, by strengthening recognition, and second by focusing on rapid restoration of renal perfusion pressure.

## Supplementary Material

sfag177_Supplemental_File
