# Supplementary material for: Acute kidney injury: incidence and quality of care in patients with type 2 diabetes
Source: Clin Kidney J. 2026 May 30;19(7):sfag177. doi: 10.1093/ckj/sfag177 (PMC13344170; doi:10.1093/ckj/sfag177)
Supplement: sfag177_Supplemental_File [file sfag177_supplemental_file.docx]

**Table 1.** Baseline characteristics of patients included in DIALECT, overall and by occurrence of in-hospital AKI episode during follow-up.
Alt text: In the whole DIALECT cohort, 39.3% of patients were female and mean age was 64 years. At baseline, RAAS-inhibitors, loop diuretics and potassium-sparing diuretics were used significantly more often in patients who developed an in-hospital AKI episode during follow-up compared to those who did not.

|  | **Data available** (n) | **Total population**  (n=672) | **Patients with in-hospital AKI** (n=106) | **Patients without in-hospital AKI** (n=566) | **P-value** |
| --- | --- | --- | --- | --- | --- |
| **Demographics** |  |  |  |  |  |
| Female sex | 672 | 264 (39.3) | 41 (38.7) | 223 (39.4) | 0.889 |
| Age, years | 672 | 64±10 | 67±9 | 63±10 | 0.004 |
| BMI (kg/m^2^) | 580 | 33±6 | 34±6 | 32±6 | 0.036 |
| Years of diabetes | 572 | 13±9 | 14±9 | 13±9 | 0.536 |
| Insulin dependence | 672 | 432 (64.3) | 79 (74.5) | 353 (62.4) | 0.016 |
| **Comorbidities** |  |  |  |  |  |
| Chronic kidney disease | 584 |  |  |  |  |
| Stage 1 |  | 213 (36.5) | 23 (25.6) | 190 (38.5) | 0.016 |
| Stage 2 |  | 233 (39.9) | 31 (34.4) | 202 (40.9) | 0.201 |
| Stage 3a |  | 61 (10.4) | 11 (12.2) | 50 (10.1) | 0.612 |
| Stage 3b |  | 63 (10.8) | 20 (22.2) | 43 (8.7) | <0.001 |
| Stage 4 and 5 |  | 14 (2.4) | 5 (5.6) | 9 (1.8) | 0.054 |
| Retinopathy | 661 | 151 (22.8) | 36 (34.0) | 115 (20.7) | 0.005 |
| Neuropathy | 670 | 261 (39.0) | 51 (48.1) | 210 (37.2) | 0.039 |
| Macrovascular diseases | 670 | 244 (36.4) | 61 (57.5) | 183 (32.4) | <0.001 |
| Coronary disease |  | 157 (23.6) | 40 (37.7) | 117 (20.9) | <0.001 |
| Cerebrovascular disease |  | 82 (12.3) | 22 (20.8) | 60 (10.7) | 0.004 |
| Peripheral disease |  | 56 (8.4) | 16 (15.1) | 40 (7.1) | 0.006 |
| **Blood pressure** |  |  |  |  |  |
| Systolic blood pressure (mmHg) | 572 | 136±16 | 138±21 | 136±15 | 0.397 |
| Diastolic blood pressure (mmHg) | 572 | 75±10 | 73±12 | 76±9 | 0.081 |
| **Drugs** |  |  |  |  |  |
| Total number of drugs | 588 | 7±3 | 8±3 | 7±3 | <0.001 |
| RAAS-inhibitors | 672 | 450 (67.0) | 87 (82.1) | 363 (64.1) | <0.001 |
| Thiazide diuretics | 672 | 208 (31.0) | 39 (36.8) | 169 (29.9) | 0.156 |
| Loop diuretics | 672 | 123 (18.3) | 34 (32.1) | 89 (15.7) | <0.001 |
| Potassium-sparing diuretics | 672 | 77 (11.5) | 21 (19.8) | 56 (9.9) | 0.003 |
| Calcium antagonists | 672 | 175 (26.0) | 31 (29.2) | 144 (25.4) | 0.413 |
| SGLT2 inhibitors | 662 | 30 (4.5) | 0 (0.0) | 30 (5.4) | 0.009 |
| **Blood and urinary values** |  |  |  |  |  |
| HbA1c (mmol/mol) | 670 | 58±12 | 59±13 | 58±12 | 0.567 |
| Albuminuria (mg/mmol creatinine) | 629 | 201 (32.0) | 45 (45.5) | 156 (29.4) | 0.002 |

BMI: body mass index.
